# Long‐term usage patterns and clinical outcomes in a community‐based differentiated antiretroviral therapy delivery programme in South Africa

**DOI:** 10.1002/jia2.26141

**Published:** 2023-07-18

**Authors:** Lara Lewis, Yukteshwar Sookrajh, Johan van der Molen, Thokozani Khubone, Munthra Maraj, Phelelani Sosibo, Rose van Heerden, Francesca Little, Reshma Kassanjee, Nigel Garrett, Jienchi Dorward

**Affiliations:** ^1^ Centre for the AIDS Programme of Research in South Africa (CAPRISA) University of KwaZulu–Natal Durban South Africa; ^2^ Department of Statistical Sciences University of Cape Town Cape Town South Africa; ^3^ eThekwini Municipality Health Unit eThekwini Municipality Durban South Africa; ^4^ Centre for Infectious Disease Epidemiology and Research School of Public Health University of Cape Town Cape Town South Africa; ^5^ Discipline of Public Health Medicine School of Nursing and Public Health University of KwaZulu‐Natal Durban South Africa; ^6^ Nuffield Department of Primary Care Health Sciences University of Oxford Oxford UK

**Keywords:** differentiated care, antiretroviral therapy, retention, viral suppression, cohort studies, HIV care continuum

## Abstract

**Introduction:**

There is little data on long‐term implementation and outcomes for people living with HIV (PLHIV) in differentiated antiretroviral therapy (ART) delivery programmes. We aimed to analyse usage patterns of and associated treatment outcomes in a community ART programme, within the Centralized Chronic Medicines Dispensing and Distribution programme, in South Africa over 3.5 years.

**Methods:**

We performed a retrospective cohort study among PLHIV on first‐line ART who were eligible for community ART delivery between October 2016 and March 2019, from 56 urban clinics in KwaZulu‐Natal, South Africa. Follow‐up ended in March 2020. We measured referral rates and, among those referred, we characterized patterns of community ART usage using group‐based trajectory modelling following referral. We used survival analysis to measure the association between community ART usage and loss‐to‐care (no visit for ≥365 days) and logistic regression to measure the association between community ART usage and viraemia (≥50 copies/ml).

**Results:**

Among the 80,801 patients eligible for community ART, the median age was 36 years, 69.8% were female and the median (interquartile range [IQR]) follow‐up time was 22 (13–31) months. In total, 49,961 (61.8%) were referred after a median of 6 (IQR 2–13) months from first eligibility. After referral, time spent in community ART varied; 42% remained consistently in community ART, 15% returned to consistent clinic‐based care and the remaining 43% oscillated between community ART and clinic‐based care. Following referral, the incidence of loss‐to‐care was 3.93 (95% confidence interval [CI]: 3.71–4.15) per 100 person‐years during periods of community ART usage compared to 5.75 (95% CI: 5.28–6.25) during clinic‐based care. In multivariable models, community ART usage was associated with a 36% reduction in the hazards of loss‐to‐care (adjusted hazard ratio: 0.64 [95% CI: 0.57–0.72]). The proportion of patients who became viraemic after first community ART referral was 5.2% and a 10% increase in time in community ART was associated with a 3% reduction in odds of viraemia (adjusted odds ratio: 0.97 [95% CI: 0.95–0.99]).

**Conclusions:**

Community ART usage patterns vary considerably, while clinical outcomes were good. Promoting consistent community ART usage may reduce clinic burden and the likelihood of patients being lost to care, while sustaining viral suppression.

## 1 INTRODUCTION

South Africa is home to over 7 million people living with HIV (PLHIV), with approximately 5.5 million accessing antiretroviral therapy (ART), making it the country with the largest population of PLHIV and ART programme globally [1]. Ensuring that ART is delivered efficiently to all PLHIV in the country remains a considerable challenge. To reduce clinic workloads and to better serve the needs of PLHIV, the World Health Organization has recommended differentiated service delivery (DSD) models for treatment [2]. These include facility‐based individual models, such as expedited clinic appointments, out‐of‐facility‐based individual models, such as community‐based ART delivery at external pick‐up points, and healthcare worker‐managed or client‐managed groups, such as adherence clubs. South Africa has adopted several of these, mainly through the Centralized Chronic Medicines Dispensing and Distribution (CCMDD) programme [3], with out‐of‐facility, individual‐based ART delivery models being widely used [4, 5]. These community‐based ART delivery programmes allow stable patients to collect pre‐packaged ART (and other chronic medication) through a variety of pick‐up points based in the community rather than from primary care clinics.

Existing evidence suggests that the receipt of community‐based ART through external pick‐up points is perceived favourably by many patients as it is more convenient and has reduced the stigma associated with receiving ART [6, 7]. However, little is known about referral rates or patterns of usage following referral. Several barriers to CCMDD implementation, such as problems with electronic prescription systems, poor distributor communication and inadequate infrastructure, have been identified [6, 7], although the impact of these on referral and usage patterns has not been quantified. Encouragingly, clinical outcomes in community‐based ART have been found to be comparable to those in clinic‐based care [8, 9]. However, findings have mostly been based on analyses using 12‐month follow‐up data.

To add to the growing body of evidence on the implementation of community‐based ART delivery, we aimed to quantify referral rates, usage patterns and clinical outcomes among patients on first‐line ART receiving community‐based ART through external pick‐up points using a large programmatic dataset with follow‐up data of up to 3.5 years.

## 2 METHODS

### 2.1 Study design and setting

We performed a retrospective cohort analysis using de‐identified data from 56 public clinics in eThekwini in KwaZulu‐Natal, South Africa. eThekwini is home to approximately 670,000 PLHIV, 74% of whom are estimated to be on ART [10]. Since 2016, national guidelines have recommended ART for all PLHIV [11]. A fixed‐dose combination of tenofovir disoproxil fumarate, emtricitabine and efavirenz was provided as the standard first‐line regimen for adults until December 2019, when tenofovir, lamivudine and dolutegravir (TLD) was introduced. Viral load is tested 6 and 12 months post ART initiation, and annually thereafter. ART is generally provided at 2‐monthly clinic appointments with a nurse. However, for patients who are stable‐in‐care, several DSD options are available. These include facility‐based individual models, facility‐ or community‐based adherence clubs and community‐based individual models providing delivery through external pick‐up points, such as networks of private pharmacies. We refer to the latter as “community ART” hereafter.

During the study period, PLHIV were eligible for community ART if they were ≥18 years, had been on the same ART regimen for ≥12 and if their two most recent viral loads (taken at least 6 months apart) were undetectable, with the most recent measurement taken within 6 months [12]. People who were pregnant, had tuberculosis (TB), uncontrolled hypertension or diabetes, or any other medical condition which required regular clinical consultations, were ineligible. Nurses or clinicians would refer eligible patients for community ART and provide 2 months ART supply. PLHIV would then have 2‐monthly ART deliveries at a pick‐up point of their choice (Figure S1). Patients would be reviewed at the clinic after 6 months and assessed for renewal of their community ART prescription. Patients could continue to visit the clinic for repeat prescriptions 6‐monthly (specifically, six 28‐day intervals) if they remained eligible. Patients would continue measuring viral loads annually, although these visits did not necessarily coincide with rescripting visits.

PLHIV receiving first‐line ART identified as eligible for community ART during the period October 2016−March 2019 were included in the cohort. A start date of October 2016 was selected because the community ART programme began in KwaZulu‐Natal from approximately mid‐2016 [13]. We excluded people who became eligible for community ART after March 2019 to allow 1 year of follow‐up before the data cut in March 2020. Patients were followed from eligibility to March 2020, or the date on which they were lost to follow‐up, transferred to another clinic or became ineligible for community ART (due to pregnancy or TB, change in ART regimen or being viraemic) if this occurred earlier. Patients who became ineligible during follow‐up were not re‐introduced into the cohort when they became re‐eligible. Since this study focused on outcomes in community ART, patients who were referred to another DSD model during follow‐up were excluded.

### 2.2 Data sources and data management

We used anonymized data from TIER.Net, an electronic register recording demographic, clinical and clinic visit data for patients receiving ART in the South African public healthcare system [14]. TIER.Net includes data on ART initiation and regimens, clinic visit dates, dates of referral into community ART, viral loads, pregnancy and TB status, and age and gender. It does not record whether patients have other medical conditions which preclude them from inclusion into community ART and, as such, these criteria were excluded from the study definition of eligibility. TIER.Net also does not contain data on community ART collection visits at external pick‐up points and, therefore, patients in community ART are only observed for their 6‐monthly clinic visits. Data were analysed using SAS, version 9.4 (SAS Institute Inc).

### 2.3 Variables

The primary outcomes were loss‐to‐care and viraemia (≥50 copies/ml). A patient was defined as being lost‐to‐care if they did not attend a clinic visit for ≥365 days. Patients documented as being transferred to another clinic within 365 days of their last observed visit were not defined as lost. We used a conservative period of 365 days for the definition of loss‐to‐care because the interval between clinic visits of those in community ART (6‐monthly) differed from that of those receiving clinic‐based ART (2‐monthly), meaning that loss‐to‐care definitions based on shorter terms were less comparable between the two groups.

The primary exposure was the use of community ART. The exposure variable was defined differently in the models of the two outcomes. For the model of loss‐to‐care, community ART use was defined as a binary variable indicating referral for community ART use at each visit. For the model of viraemia, community ART usage was defined as the proportion of days spent in community ART since the last viral load was measured, as viral loads are likely affected by both medium‐term and short‐term behaviour. Other variables considered as potential confounders of the association between the outcomes and exposure were age, gender, time since initiation on ART and year of first referral into community ART.

### 2.4 Statistical analysis

The overall community ART referral percentage was calculated as the proportion of eligible clients in the cohort that were ever referred before the end of follow‐up in March 2020. Referral percentages were also disaggregated by the clinic. Monthly referral percentages were calculated as the proportion of eligible patients who had not been previously referred for community ART, who were referred in that month. All subsequent analyses, namely the analysis of community ART usage patterns and the association between community ART and clinical outcomes of loss‐to‐care and viraemia, were performed using data from patients referred into community ART before March 2019, from their point of referral.

A swimmer's plot illustrating each patient's movement between community ART and clinic‐based care after referral was created. Group‐based trajectory modelling (GBTM), extended for non‐random subject attrition [15], was used to identify subgroups of patients with similar patterns of community‐ART use. Competing models, using a polynomial of order 3 for the profile over time, with 2–4 subgroups were fit and compared using Bayesian Information Criterion. Average posterior probabilities of group membership were also assessed using a level of 0.8 as a suitable cut‐off. Data from the first 6 months in community ART were not used in the GBTM as all patients should have remained in community ART until their first renewed prescription 6 months after referral.

The association between community ART use and loss‐to‐care was modelled using Cox regression and time 0 was set at the date of first referral. Retained patients were censored on 1 March 2019 (the last date on which 365 days of attendance before 1 March 2020 could be observed), or when they were ineligible or transferred to another clinic. For patients who became lost to care, the exact date of loss‐to‐care was unknown, although we presumed that it occurred between a patient's last observed visit and their next expected visit. For patients in clinic‐based care, the next scheduled visit was generally after 56 days, and so we imputed the loss‐to‐care date as the last observed visit date plus a random number between 1 and 56 (2*28) days (Figure S2). Similarly, for patients in community ART, the next scheduled visit was generally after 168 days, and so we imputed the date of loss‐to‐care as the last visit date plus a random number between 1 and 168 (6*28) days. A frailty model term was included to incorporate clinic correlation. The multivariable model included patient age, gender, time on ART and year of referral (2018 and 2019 data were combined as there were only 2 months of data from 2019).

For the analysis of the association between community ART use and viraemia, survival analysis was not deemed appropriate as viral loads were measured approximately annually. Instead, an indicator of viraemia measured over time was modelled using logistic regression with generalized estimating equations accounting for clustering by the clinic. The community ART variable measuring the proportion of days spent in community ART since the last viral load was categorized into 10% intervals and treated as continuous. The multivariable model included age, gender, time on ART in years and the number of months between viral loads.

Three sensitivity analyses were performed. To test the impact of informative censoring in the Cox regression introduced by the censoring of patients becoming viraemic during follow‐up, all patients censored for becoming viraemic were assumed to become lost to care immediately after their point of viraemia. The approach was informed by the assumption that those becoming viraemic have a higher risk of becoming lost to care. To understand whether incomplete or infrequent viral load measurements impacted our findings, both regressions were re‐run using a subset of patients whose viral loads between referral and end of follow‐up occurred approximately annually.

### 2.5 Ethical approval

This work was approved by the University of Kwazulu‐Natal Biomedical Research Ethics Committee (BE646/17), the KwaZulu‐Natal Department of Health's Provincial Health Research Ethics Committee (KZ_201807_021) and the eThekwini Municipality Health Unit, with a waiver for informed consent for analysis of anonymized, routinely collected data.

## 3 RESULTS

### 3.1 Cohort characteristics and referral rates

**Figure 1:**
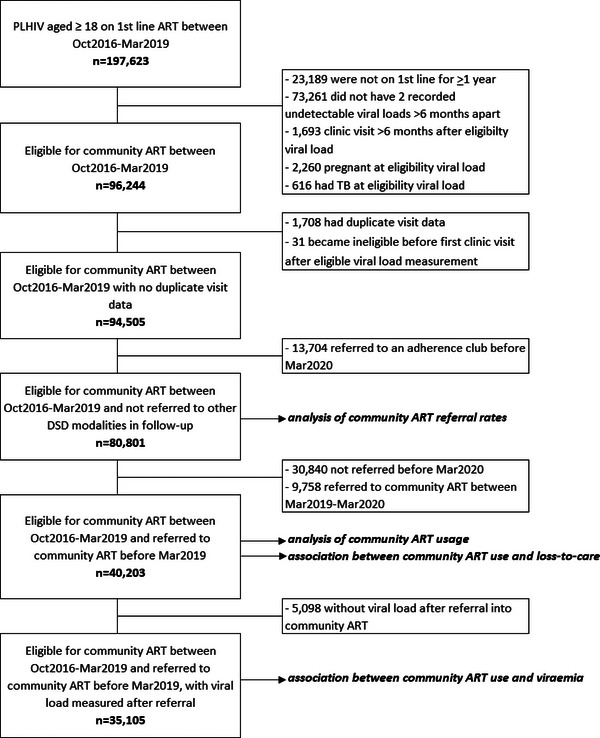
Flow diagram of the number of PLHIV on first‐line ART, eligible for community‐based ART through the CCMDD programme between October 2016 and March 2019 who received community‐based ART delivery or clinic‐based care between October 2016 and March 2020, and sub‐samples used in the study analyses.

Between October 2016 and March 2019, 197,623 PLHIV received first‐line ART, of whom 96,244 (49%) were eligible for referral into community ART (Figure 1). Among ineligible patients, 23% had been on ART for less than 1 year, 73% did not meet viral load eligibility criteria, 2% were pregnant and less than 1% had TB. A further 1739 people were removed due to having duplicate visit data in follow‐up or because they became ineligible before their first clinic visit after viral load eligibility. An additional 13,704 patients were excluded because they attended an adherence club during follow‐up. In the remaining 80,801 patients, the median follow‐up time from eligibility to the last clinic visit before March 2020 was 22 (interquartile range [IQR] 13–31) months. The median (IQR) age at eligibility was 36 (31–43) years, 70% were female and 98% were on a TEE regimen (Table 1).

**Table 1:** Baseline characteristics of patients on first‐line ART at time of meeting eligibility for the community ART programme between October 2016 and March 2019

		Total (*N* = 80,801)	Referred to community ART (*N* = 49,961)	Remained in clinic (*N* = 30,840)
Age, median (IQR)	Female	36 (31−43)	37 (31−43)	35 (30−43)
Gender, % (*n*)		69.8 (56,415)	70.5 (35,202)	68.8 (21,213)
Months on first‐line ART, median (IQR)		27 (14−52)	32 (15−58)	24 (13−48)
ART regimen at eligibility	TEE	98.0 (79,223)	98.5 (49,227)	97.3 (29,996)
	Other	2.0 (1578)	1.5 (734)	2.7 (844)
Most recent CD4 count^a^, % (*n*)	>500	32.3 (26,080)	32.8 (16,412)	31.3 (9668)
	351−500	18 (14,550)	18 (9004)	18 (5546)
	201−350	13.9 (11,200)	13.3 (6624)	14.8 (4576)
	< = 200	6.8 (5528)	5.5 (2753)	9 (2775)
	Missing	29 (23,443)	30.4 (15,168)	26.8 (8275)
Year of eligibility, % (*n*)	2016	20.6 (16,684)	21.5 (10,749)	19.2 (5935)
	2017	42.3 (34,216)	45.1 (22,538)	37.9 (11,678)
	2018	31.7 (25,634)	29.1 (14,534)	36 (11,100)
	2019	5.3 (4267)	4.3 (2140)	6.9 (2127)
Months to community ART referral, median (IQR)		n/a	6 (2−13)	n/a

^a^ Measured no more than 2 years before eligibility or 6 months afterwards.

**Figure 2:**
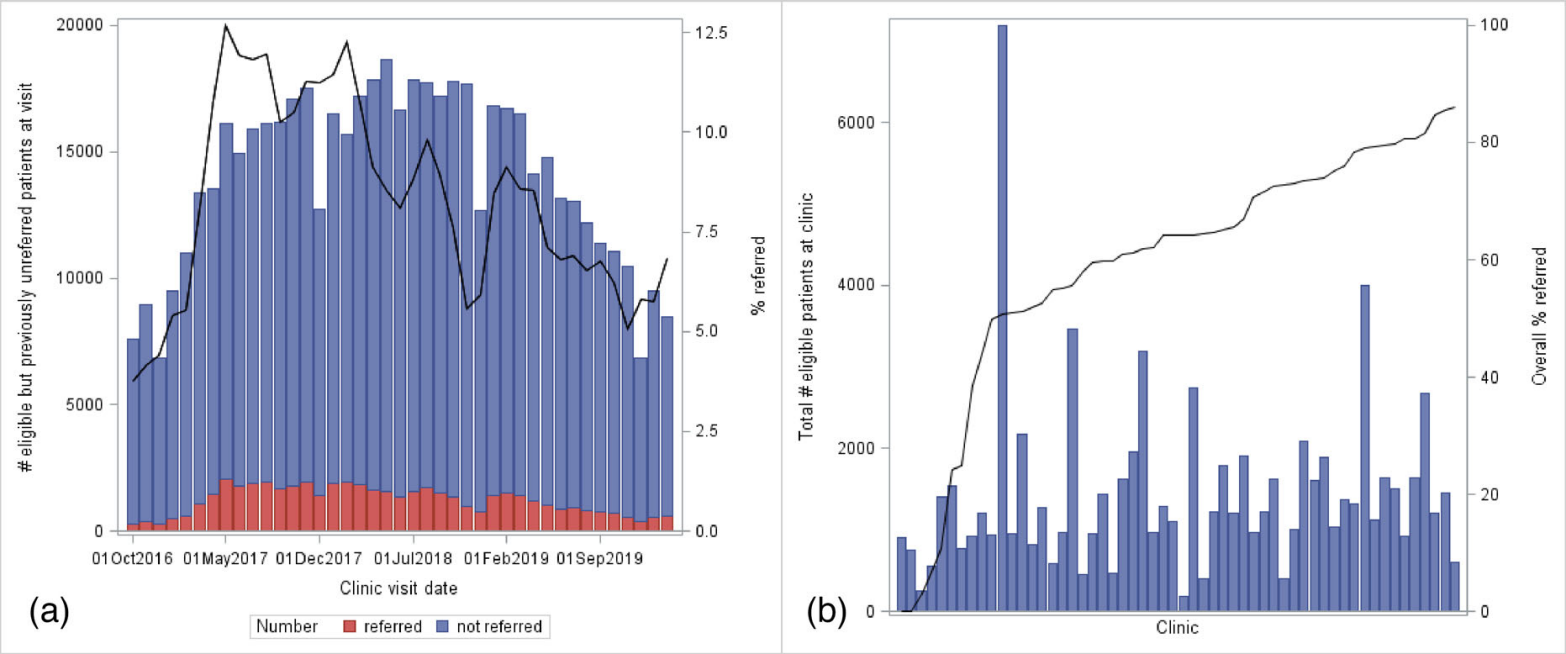
Community ART referral rates (*N* = 80,801). (a) Percentage of eligible but previously unreferred patients referred into community ART in each month and (b) total percentage of eligible patients referred into community ART by clinic.

Of the 80,801 patients eligible for community ART, 49,961 (62%) were referred before the end of follow‐up, with a median (IQR) time to referral from eligibility of 6 (2–13) months (Table 1). The characteristics of those referred were similar to those who, while eligible, were not referred, except with respect to time on ART, with those referred into community ART having a longer median time since ART initiation. Monthly referral rates among eligible patients who had never previously been referred varied between 5% and 12.5% between October 2016 and March 2020 (Figure 2a). The percentage of patients referred varied by clinic, with some clinics referring no patients and others referring up to 86% (Figure 2b).

### 3.2 Patterns of community ART use

**Figure 3:**
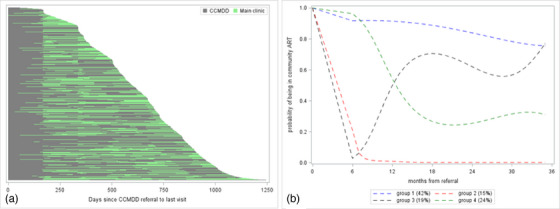
Patterns of community ART use among patients on first‐line ART from first referral to last observed visit (*N* = 40,203). (a) Swimmer's plot of time in community ART (grey) and time in clinics (green) following referral, and (b) groups of patients identified through GBTM as having common community ART exposure trajectories.

Among 40,203 patients referred to community ART before March 2019, many moved between community ART and clinic‐based care following referral despite remaining eligible for community ART (Figure 3a). The median (IQR) proportion of time spent in community ART after referral was 77% (55%–95%). GBTM was used to identify clusters of patients with similar community ART usage patterns and a four‐group model was selected (Figure 3b and Table S1). Approximately 42% of all patients consistently attended community ART (group 1). A second group, comprising of 15%, left community ART at their first script renewal and were not rescripted into the programme thereafter. The third group, making up 19% of participants, also left community ART after their first script renewal but were later rescripted and had a high probability of remaining in the programme thereafter. In the final group, comprising 24% of all referrals, patients had a low probability of using community ART from approximately 12 months after the first referral. We ran a post‐hoc analysis using a chi‐square test to explore whether the timing of the first viral load due after community ART referral was associated with the proportion of patients leaving community ART at their first clinic visit after referral. Fifty‐two percent of patients with an annual viral load due within 3 months of their first clinic visit after referral left community ART compared to 36% among those due a viral load more than 3 months later (*p*<0.01).

### 3.3 Loss‐to‐care

The incidence rate of loss‐to‐care after community ART referral was 4.35 (95% confidence interval [CI]: 4.15–4.56) per 100 person‐years. Higher rates of loss‐to‐care were observed during periods of clinic‐based care compared to periods of community ART (5.75 [5.28–6.25] vs. 3.93 [3.71–4.15] per 100 person‐years, respectively). In the multivariable Cox regression, the hazard of loss‐to‐care was lower in community ART than in clinic‐based care (adjusted hazard ratio [aHR]: 0.64 [95% CI: 0.57–0.72], Table 2). The sensitivity analysis assessing the impact of possible informative censoring showed a similar association between community ART use and the combined outcome of loss‐to‐care and viraemia (aHR: 0.73 [95% CI: 0.66–0.81], Table S2). Lastly, a sensitivity analysis only including patients who had viral load values for every year of follow‐up (*N* = 14,288) confirmed a negative association between community ART use and hazard of loss‐to‐care (aHR: 0.51 [95% CI: 0.40–0.66]) (Table S2).

**Table 2:** Association between community ART exposure and loss‐to‐care among patients referred to community ART between October 2016 and March 2019 (*N* = 40,203)

	Hazards ratio (95% CI)	Adjusted hazards ratio (95% CI)
Age in years at referral	0.97 (0.97−0.98)	0.98 (0.97−0.98)
Gender (female vs. male)	0.77 (0.65−0.85)	0.72 (0.65−0.79)
Years on first‐line ART at referral	0.90 (0.88−0.92)	0.93 (0.91−0.95)
Year of eligibility (2016 vs. 2018/2019)	0.62 (0.47−0.83)	0.64 (0.48−0.85)
(2017 vs. 2018/2019)	0.81 (0.72−0.90)	0.84 (0.75−0.93)
Exposure to community ART (yes vs. no)	0.64 (0.57−0.72)	0.64 (0.57−0.72)

### 3.4 Viraemia

Among the 40,203 patients referred for community ART, 35,105 (87%) had at least one viral load measured after referral and were included in the analysis of the association between community ART use and viraemia. Of these, 1827 (5.2%) became viraemic during follow‐up. An increase in the amount of time spent using community ART instead of clinic‐based ART delivery since the last viral load was associated with lower odds of viraemia (adjusted odds ratio [aOR]: 0.97 [95% CI: 0.97–0.99] for a 10% increment in time spent using community ART, Table 3). In a sensitivity analysis only including patients with viral loads measured approximately annually (*N* = 13,898), there was no evidence of association between time spent using community ART and viraemia (aOR: 0.98 [95% CI: 0.95–1.01]) (Table S3).

**Table 3:** Association between community ART exposure and viraemia (VL≥50 copies/ml) among patients referred to community ART between October 2016 and March 2019 (*N* = 35,103)

	Odds ratio (95% CI)	Adjusted odds ratio (95% CI)
Age in years at referral (20−29 vs. 60+)	1.35 (0.88−2.07)	1.59 (1.04−2.41)
(30−39 vs. 60+)	1.02 (0.83−1.24)	1.14 (0.93−1.39)
(40−49 vs. 60+)	1.01 (0.82−1.25)	1.06 (0.86−1.31)
(50−59 vs. 60+)	1.13 (0.92−1.39)	1.15 (0.93−1.42)
Gender (female vs. male)	0.78 (0.72−0.86)	0.78 (0.71−0.86)
Time on ART (years)	1.02 (1−1.04)	1.02 (1.0−1.04)
Months since last viral load	1.02 (1.01−1.03)	1.02 (1.01−1.03)
% exposure to community ART in 12 months preceding viral load (10% increment)	0.97 (0.96−0.99)	0.97 (0.95−0.99)

## 4 DISCUSSION

Using data from a large retrospective cohort of patients on first‐line ART, we assessed referral rates, usage patterns and clinical outcomes of community‐based ART delivery through external pick‐up points over a period of 3.5 years. We found that many patients eligible for community ART were not referred into the programme, and among those referred, usage patterns were inconsistent. The variability in usage allowed us to assess the association between programme use and clinical outcomes using data from referred patients only, thus reducing issues of non‐comparability arising from comparing outcomes of those referred to outcomes among those never referred. The findings suggest that community ART enhances retention‐in‐care and sustains viral suppression among first‐line patients in the long term.

There is growing evidence asserting that, among stable patients, using community ART is as effective in maintaining viral suppression and retention‐in‐care as clinic‐based care [8, 9]. In a nationwide cluster‐randomized evaluation of community ART delivery in South Africa, 12‐month retention (<90 days late for a visit, alive and not transferred) and viral suppression (<400 copies/ml) among those with viral loads was 82% and 95%, respectively, and found to be comparable to outcomes observed in eligible patients who were not referred [16]. To date, little research has examined outcomes in community ART programmes beyond 12 months. However, here we show that community ART use is associated with good clinical outcomes over a period of up to 3.5 years. We also show higher retention during periods accessing community ART than in periods accessing ART at clinics. Long clinic queues, transportation costs and travel times cited by patients as deterrents to attending clinics could account for the differences in retention‐in‐care observed in this study. In addition, some patients perceive referral into community ART as a reward for good adherence [6], making them more motivated to adhere to treatment, and remain engaged in care.

We also illustrate that patterns of community ART usage vary considerably among patients. There are few studies reporting data on community‐ART usage patterns. An Eswatini study which assessed three community ART delivery models reported that 18% of patients left community ART after 12 months [17]. In our setting, only 42% of patients remained consistently in community ART during follow‐up, while 15% left permanently after their first rescripting visit. The remaining 43% oscillated between community and clinic‐based ART delivery. We are unable to determine the reasons for attrition using our data. A qualitative evaluation of the CCMDD implementation in South Africa identified several possible reasons for programme attrition, including patient, provider and distributor communication errors, administrative errors, inflexible CCMDD policies around re‐fill dates or pick‐up points and patient choice [7]. The authors also noted clinic computing and staffing constraints which may partly explain the jump in attrition at rescripting visits observed in this study. We explored whether a short (<3‐month) lag between the timing of the annual viral load and the first clinic visit after referral (resulting in patients returning to clinic‐based ART delivery until their viral load result had been received) may have contributed to the attrition from community ART observed in this study. We found that there was a higher probability of patients returning to clinic‐based ART delivery at their first clinic visit after programme referral if they were due a viral load test soon after this visit. Updated guidance from the South African Department of Health recommends that annual viral loads be completed with community ART rescripting visits, and that contact tracing should be conducted for those patients found to be viraemic [11]. More data are required to assess the impact of this change on retention in community ART.

These findings have several implications for policy and research. The number of patients eligible for community‐based ART far exceeds the number referred. Barriers to uptake need to be understood and quantified, and the feasibility of scaling‐up implementation to reach willing, yet unreferred, patients needs to be assessed. Future studies examining clinical outcomes in DSD models should consider programme retention among referred patients when interpreting results. The reasons for breaks in rescripting after referral into the programme need to be researched, and programme changes that will facilitate the timeous rescripting of patients performing well in the community‐based ART need to be implemented. The impact of the shift to TLD as the standard first‐line regimen on these findings should be investigated, as the improved tolerability profile of dolutegravir [18] may assist in lowering the rates of attrition from community ART observed here. Finally, these findings should be confirmed in post‐COVID‐19‐pandemic conditions.

The study has limitations. In South Africa, the length of ART dispensed is typically no more than 2 months, and thus our results may not generalize to settings where a longer supply is available. We may have underestimated referral rates due to the exclusion of data on uncontrolled chronic conditions from the study definition of eligibility. Reassuringly, however, the overall referral rate was similar to that published previously [19]. We also used a conservative definition of loss‐to‐care based on 365 days, and as such were unable to compare short‐term interruptions in care between the two groups. Limitations associated with using TIER.Net data may have affected our findings. First, although retention rates were high, they were likely underestimated as silent transfers between clinics could not be identified. Viral load data may have been incomplete, and because we worked with a large de‐identified dataset, it was not possible to find missing viral loads in the South African National Health Laboratory Service database. Thus, we likely failed to censor some patients who became viraemic during follow‐up. To estimate the impact of this, we conducted sensitivity analyses using patients with annual viral load data. The results supported the conclusion that community ART use was associated with lower rates of loss‐to‐care, but the association with reduced viraemia was no longer significant (although of a similar magnitude). Finally, while our estimates of association adjusted for possible confounders collected in TIER.Net, we could not adjust for unmeasured confounders.

## 5 CONCLUSIONS

In conclusion, using follow‐up data of up to 3.5 years, we demonstrate that the use of community ART delivery through external pick‐up points is associated with better retention and similar viral suppression rates compared to clinic‐based ART delivery among PLHIV on first‐line ART. However, rates of referral into community ART in this setting were sub‐optimal, and usage patterns following referral varied considerably. To maximize the impact of community ART programmes on patient outcomes and clinic operations, efforts to support timeous referrals and continuity of care in these programmes among eligible patients choosing this DSD modality should be enhanced.

## COMPETING INTERESTS

### AUTHORS’ CONTRIBUTIONS

JD, NG and LL conceived the analysis. TK, PS, YS, MM and RvH oversaw data collection. TK and JvdM oversaw data curation. TK, JvdM, LL and JD have verified the underlying data. LL, JvdM and JD analysed the data with inputs on design and implementation from RK and FL. LL drafted the manuscript. All authors critically reviewed and edited the manuscript and consented to final publication.

## FUNDING

This work was supported, in whole or in part, by the Bill & Melinda Gates Foundation [INV‐051067]. Under the grant conditions of the Foundation, a Creative Commons Attribution 4.0 Generic License has already been assigned to the Author Accepted Manuscript version that might arise from this submission. This work was also supported by a COVID‐19 Adaptations to Differentiated Service Delivery grant from the International AIDS Society, and a Fast Track Cities Implementation Science grant from the International Association of Providers of AIDS Care (IAPAC) (2021‐ISG‐Y1‐10004). JD is supported by the Wellcome Trust (grant number 216421/Z/19/Z). For the purpose of open access, the author has applied a CC BY public copyright licence to any Author Accepted Manuscript version arising from this submission.

## Supporting information

Supporting information

## Data Availability

The data used for this analysis cannot be shared publicly because of legal and ethical requirements regarding the use of routinely collected clinical data in South Africa. Researchers can request access to the data from the eThekwini Municipality Health Unit and the South African National Department of Health TB/HIV Information System (contact details obtainable upon request to JD).
