# Supplementary material for: Long‐term usage patterns and clinical outcomes in a community‐based differentiated antiretroviral therapy delivery programme in South Africa
Source: J Int AIDS Soc. 2023 Jul 18;26(7):e26141. doi: 10.1002/jia2.26141 (PMC10354003; doi:10.1002/jia2.26141)
Supplement: Supplementary file 1 — Supporting information [file JIA2-26-e26141-s001.docx]

## **Supplementary Files**

**Figure *S*1: Clinic visit schedule by ART delivery model in South Africa. Visits to community pick-up points not observed in TIER.Net data**


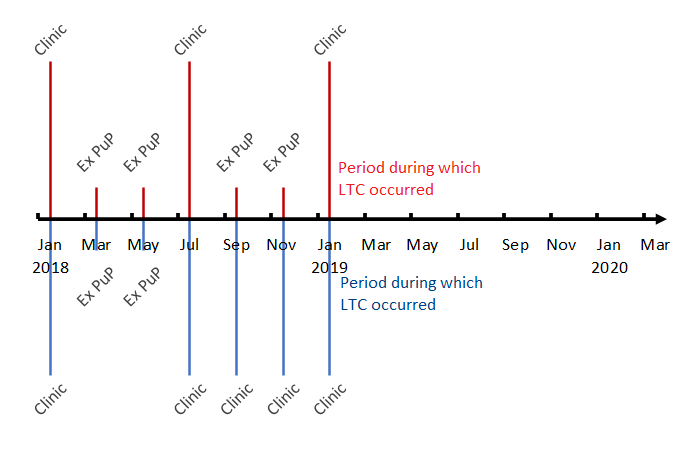


**Figure *S*2: Illustration of paths to loss-to-care and loss-to-care event intervals among patients referred for community ART delivery who (1) remained in community ART (red) or (2) returned to standard of care (SOC) and were last seen while in SOC (blue). Community ART collections occurred at external pick-up points (Ex PuP) and these visits were not captured in TIER.Net.**

**Table *S*1: Maximum likelihood estimates from the group-based trajectory model with drop-out**

| Group | Parameter | Estimate | p-value |
| --- | --- | --- | --- |
| 1 | Intercept | 2.40 | <0.001 |
|  | Linear | 0.03 | 0.0077 |
|  | Quadratic | -0.001 | <0.001 |
|  | Cubic | 0.0001 | <0.001 |
|  | Drop-out | -1.90 | <0.001 |
| 2 | Intercept | -1.32 | <0.001 |
|  | Linear | -1.76 | <0.001 |
|  | Quadratic | 0.32 | <0.001 |
|  | Cubic | -0.02 | <0.001 |
|  | Drop-out | -2.28 | <0.001 |
| 3 | Intercept | -3.56 | <0.001 |
|  | Linear | 0.89 | <0.001 |
|  | Quadratic | -0.06 | <0.001 |
|  | Cubic | 0.001 | <0.001 |
|  | Drop-out | -2.24 | <0.001 |
| 4 | Intercept | 3.24 | <0.001 |
|  | Linear | -0.71 | <0.001 |
|  | Quadratic | 0.04 | <0.001 |
|  | Cubic | -0.001 | <0.001 |
|  | Drop-out | -2.07 | <0.001 |
|  |  |  |  |

## **Table S2: Results from the sensitivity analyses for loss-to-care. Association between (A) community ART exposure and combined loss-to-care and viraemia (N=40,203) and (B) between community ART exposure and loss-to-care among patients with annual viral load data (N=14,288)**

|  | Adjusted hazards ratio (95% CI) | |
| --- | --- | --- |
|  | A | B |
| Age in years at referral | 0.98(0.98-0.99) | 0.98(0.97-0.99) |
| Gender (Female vs male) | 0.77(0.70-0.84) | 0.72(0.58-0.91) |
| Years on first-line ART at referral | 0.94(0.92-0.96) | 0.99(0.94-1.04) |
| Year of eligibility (2016 vs 2018/2019) | 0.82(0.64-1.05) | 0.56(0.29-1.19) |
| (2017 vs 2018/2019) | 0.93(0.84-1.03) | 0.73(0.55-0.95) |
| Exposure to community ART (Yes vs No) | 0.73(0.66-0.81) | 0.51(0.40-0.66) |

## **Table S3: Results from the sensitivity analyses for viraemia. Association between community ART exposure and viraemia (VL>50 copies/mL) among patients with annual viral load data (N=13,898)**

|  | Adjusted odds ratio (95% CI) |
| --- | --- |
| Age in years at referral (20-29 vs 60+) | 1.83(0.95-3.51) |
| (30-39 vs 60+) | 1.47(1.01-2.14) |
| (40-49 vs 60+) | 1.35(0.94-1.95) |
| (50-59 vs 60+) | 1.51(1.03-2.22) |
| Gender (Female vs male) | 0.72(0.62-0.83) |
| Time on ART (years) | 1.02(0.99-1.04) |
| Months since last viral load | 0.95(0.87-1.03) |
| % exposure to community ART in 12 months  preceding viral load (10% increment) | 0.98(0.95-1.01) |
